# Molecular detection of bacteria in plant tissues, using universal 16S ribosomal DNA degenerated primers

**DOI:** 10.1080/13102818.2014.937139

**Published:** 2014-10-22

**Authors:** Georgios Tsoktouridis, George Tsiamis, Nikolaos Koutinas, Sinclair Mantell

**Affiliations:** ^a^ Department of Agricultural Sciences, Imperial College at Wye, University of London, Ashford, Kent, UK; ^b^ Department of Crop Production, School of Agriculture, Alexander Technological Educational Institute of Thessaloniki, Thessaloniki, Greece; ^c^ Department of Environmental and Natural Resources Management, University of Western Greece, Agrinio, Greece; ^d^ Nakhlatec International Development Advisors, Gödelöv, Genarp, Sweden

**Keywords:** PCR, diagnostics, bacteria, plant tissues, 16S rDNA, degenerated primers

## Abstract

Highly specific, sensitive and rapid tests are required for the detection and identification of covert bacterial contaminations in plant tissue cultures. Current methods available for this purpose are tedious, time consuming, highly error prone, expensive, require advanced technical expertise and are sometimes ineffective. We report here the development of a sensitive polymerase chain reaction (PCR) based method for the rapid detection and identification of bacteria occurring in plant tissue cultures. A total of 121 16S ribosomal DNA (rDNA) coding regions from 14 different groups of bacteria, algae and plants, available in the *Gene Bank/European Molecular Biology Laboratory* databases, were aligned and several conserved DNA sequences of bacterial origin were identified. From those, five degenerated primers were designed in order to amplify only the bacterial DNA present in mixed plant/bacteria genomic DNA extracts. A known amount of bacterial suspension of either covert *Pseudomonas* or covert *Bacillus* were added to *in vitro* plant leaves and total plant/bacterial DNA extracted using three different methods to determine the lowest number of bacteria required to be present in order to allow their detection. The highest sensitivity of the bacterial cell detection was 2.5 × 10^6^ cells of both *Bacillus* and *Pseudomonas* inoculums, using template DNA prepared by the *MiniPrep* method. Generation of PCR amplification fragments was achieved only for the 16S rDNA bacterial gene by using four combinations of degenerated primers. Successive sequence analysis of these amplified fragments led to the rapid detection and molecular identification of bacteria covertly associated with plants.

## Introduction

Plant cell and tissue culture is an important tool for the aseptic production of cells, tissues and organs in both basic and applied research. It has been extensively employed in commercial production of plant metabolites, biotransformation of pharmaceuticals, production of proteins including antibiotics, plant genetic manipulation and massive production of plants in the horticultural industry.[1] It is very important that *in vitro* cultures are free of biological contamination and are maintained as aseptic cultures during manipulation, growth and storage. Contamination management is focused on the elimination of micro-arthropods, fungi, bacteria and viruses/viroids.[2] The danger of contaminants is well known in the majority of commercial and scientific plant tissue culture laboratories and losses due to contamination still average between 3% and 15% at every subculture. Most of these constantly occurring losses are caused by fungal, yeast, and bacterial contaminants.[3] Roughly 20%–55% of contamination losses to *in vitro* plant cultures are caused by bacteria.[4] Furthermore, loss of valuable research material in micropropagation is frequently caused by endophytic pathogenic bacteria and viruses. Despite the awareness of the contamination problem, many laboratories are still troubled by sudden outbreaks of bacterial infestations resulting in the loss of culture stocks that had been free of visible contamination for long periods of time.[2]

Identification of microorganisms by conventional laboratory tests takes only a few days and that by molecular approaches reduces the identification time to hours. The emergence of DNA diagnostics is revolutionizing the whole approach in identifying and monitoring microbes. Such methods use genotypic rather than phenotypic markers to identify specific microbes and the strengths of DNA diagnostics lie in the fact that: (1) nucleic acids can be rapidly and sensitively measured and (2) the sequence of nucleotides in a given DNA molecule is sufficiently specific to be used for reliable diagnosis. Similar diagnostic studies have been exclusively and specifically developed for anammox bacteria in soils [5] and for identifying the genetic diversity of 60 *Methylobacterium* spp. strains from eight different host plants.[6] Both diagnostic studies target the 16S ribosomal gene of bacteria. Identification of bacteria isolated from *in vitro* cultures of *Billbergia magnifica* ssp. *acutisepalia* revealed many important species such as *Bacillus cereus*, *Bacillus thuringiensis*, *Bacillus fusiformis*, *Agrobacterium* sp., *Microbacterium* sp., *Sphingomonas* sp., *Pseudomonas putida* and *Paenibacillus amylolyticus*, which were identified by sequencing of the 16S ribosomal DNA (rDNA) gene.[7]

In this study, we describe a polymerase chain reaction (PCR) based strategy and associated protocols that allow the rapid detection of two bacterial contaminants in tissue-cultured plants of the ornamental plant *Billbergia* by amplifying only a fragment of the 16S rDNA gene of the bacteria and not the conserved region of the 16S ribosomal gene of cpDNA. This PCR product can be subsequently used for classification of the detected bacteria by cloning and sequencing.[8]

## Materials and methods

### Alignment of 16S rRNA sequences of different organisms

In order to identify conserved regions of the 16S rDNA gene in bacteria and the prokaryotic DNA of plants, DNA sequences of the 16S region belonging to (1) 79 bacteria of known phyla, (2) 10 uncultured bacteria, (3) 10 algae and (4) 22 plants (Table 1) were aligned using the ClustalX from the DNAStar Nucleotide Sequence Analysis Package. The 16S rDNA sequences were obtained from the National Center for Biotechnology Information (NCBI) database.

**Table 1.** List of organisms of the 16S rDNA sequences aligned and accession numbers.

Organism^a^	Accession no.^b^	Organism^a^	Accession no.^b^
**W_1_ – Proteobacteria**		*Mycoplasma edwardii*	U73903
*Acetobacter aceti*	X74066	*Propionibacterium freudenreichi*	X53217
*Agrobacterium* sp.	AB006037	*Streptococcus caprinus*	Y10869
*Azotobacter vinelandii*	L40329	*Thermoactinomyces dichotomicus*	L16902
*Beggiatoa* sp.	AF035956	**W_11_*– Thermotoga–Thermosipho***	
*Chromatium tepidum*	M59150	*Thermosipho melanesiensis*	Z70248
*Escherichia coli*	J01859	*Thermotoga elfii*	X80790
*Pseudomonas* sp.	U81871	*Thermotoga subterr*anea	U22664
*Hyphomicrobium vulgare*	X53182	**W_12_ – *Aquifex–Hydrogenobacter***	
*Myxococcus xanthus*	M34114	*Aquifex pyrophilus*	M83548
*Neisseria weaveri*	L10738	*Hydrogenobacter acidophilus*	D16296
*Nitrosomonas communis*	Z46981	*Hydrogenobacter thermophilus*	Z30189
*Pseudomonas lemoignei*	X92554	**Uncultured bacteria**	
*Rhizobium leguminosarum* bv. *trifolii*	U31074	*Uncultured bacterium* A11	AF125199
*Rhodopseudomonas palustris*	D25312	*Uncultured bacterium* BH017	AF052412
*Vibrio* sp.	X97988	*Uncultured bacterium* D006	AF125201
**W_2_ – Green sulphur bacteria**		*Uncultured bacterium* D084	AF125200
*Chlorobium vibrioforne*	Y10649	*uncultured gamma proteobacterium*	AF114506
**W_3_ – Green non-sulphur bacteria**		**400m-ATT-1**	
*Chloroflexus aurantiacus*	M34116	*uncultured Planctomyces* clone 7F15	AF029079
*Herpetosiphon* sp.	X86447	*uncultured Pirellula* clone 5H12	AF029076
*Thermomicrobium roseum*	M34115	*uncultured eubacterium* env.OPS 17	AF018199
**W_4_** – **Cyanobacteria**		*uncultured bacterium* SJA-176	AJ009504
*Chlorogloeopsis* PCC7518	X68780	**Archaea**	
*uncultured cyanobacterium* WH12	AJ007375	*Archaeoglobus fulgidus*	Y00275
*Microcystis aeruginosa*	AB008323	*Desulfurococcus mobilis*	M36474
*Synechococcus* PCC7002	AJ000716	*Haloarcula* sp.	AB010965
**W_5_ – *Planctomyces–Pirella***		*Methanobacterium subterraneum*	X99045
*Pirellula clone* 5H12	AF029076	*Methanococcus vulcanus*	AF051404
*Pirellula marina*	X62912	*Methanosarcina mazei*	AF028691
*Planctomyces* sp.	X85249	*Methanospirillum hungatei*	M60880
**W_6_ – *Spirochetes***		*Pyrodictium occultum*	M21087
*Borrelia hispanica*	U42294	*Sulfolobus solfataricus*	D26490
*Leptospira biflexa*	Z98591	*Thermococcus* sp.	Y08384
*Spirochaeta africana*	X93928	*Thermoplasma acidophilum*	M38637
**W_7_ – *Bacteroides–Flavobacterium***		*Thermoproteus tenax*	M35966
*Bacteroides* sp.	AF070444	**Algae**	
*Cytophaga aprica*	D12655	*Antithamnion* sp.	U03555
*Saprospira grandis*	M58795	*Costaria costata*	X53229
*Flavobacterium balustinum*	D14016	*Chlorarachnion* CCMP240	U21491
*Flavobacterium branchiophilum*	D14017	*Chlorarachnion reptans*	U21490
*Flavobacterium breve*	D14022	*Cyanidium caldarium*	AF022186
*Flavobacterium indologenes*	X67848	*Guillardia theta*	X06428
*Flavobacterium* sp.	AJ009687	*Mallomonas striata*	M87333
*Flavobacterium meningosepticum*	D14018	*Stephanopyxis broschii*	M87330
*Flavobacterium odoratum*	D14019	*Synura spinosa*	M87336
*Flavobacterium salegens*	M92279	*Tribonema aequale*	M55286
*Flavobacterium yabuuchiae*	D14021	**Plants–chloroplast**	
**W_8_ – *Chlamydia***		*Alnus incana*	X54299
*Chlamydia pecorum*	AB001777	*Chlamydomonas reinhardtii*	X03269
*Chlamydia* sp.	D88317	*Chlorella vulgaris*	AB001684
*Chlamydia psittaci*	U73108	*Daucus carota*	X78534
*Chlamydia trachomatis*	AE001347	*Nicotiana plumbaginifolia*	X70938
**W_9_** – ***Deinococcus–Thermus***		*Carica papaya*	U76911
*Deinococcus geothermalis*	Y13040	*Epifagus virginiana*	M81884
*Deinococcus murrayi*	Y13043	*Euglena gracilis*	X70810
*Thermus* sp.	AB020888	*Glycine max*	AF041468
*Thermus thermophilus*	M26923	*Marchantia polymorpha*	X04465
**W_10_ – Gram-positive bacteria**		*Odontella sinensis*	Z67753
*Actinomyces* sp.	Y13025	*Orobanche minor*	AJ007728
*Actinoplanes philippinensis*	U58525	*Oryza sativa*	X15901
*Arthrobacter* sp.	X93356	*Pinus thunbergii*	D17510
*Bacillus* sp.	AB020200	*Pisum sativum*	X55033
*Clostridium* sp.	Y12289	*Porphyra purpurea*	U38804
*Corynebacterium auris*	X81873	*Prototheca wickerhamii*	AJ222802
*Lactobacillus pontis*	X76329	*Pteridium aquilinum*	Z81323
*Micrococcus roseus*	X87756	*Sinapis alba*	M15915
*Mycobacterium* sp.	AJ012626	*Spirogyra maxima*	U24596
		*Toxoplasma gondii*	X65508
		*Zea mays*	X86563

^a^ **W_1_–W_12_** are groups of bacteria according to Woese's classification system.[8]

^b^Accession number of 16S rDNA sequence for electronic retrieval from database.

### Design of oligonucleotide primers

**Figure 1.**
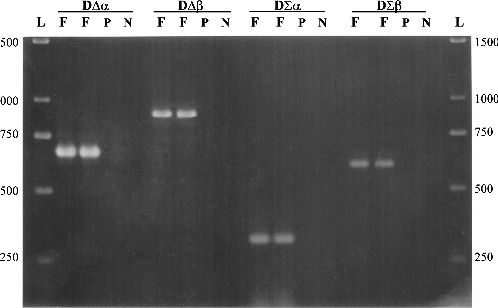
Detection of bacterial DNA in plant tissues, using four sets of primers. F is an amplified fragment (in duplicated reactions) at different positions of the bacterial 16S rDNA gene. P is the control template using pure plant DNA and N is the negative control with no DNA template. L is 1 kb ladder (GeneRuler^TM^, MBI Fermentas, Lithuania).

To amplify only fragments of bacterial 16S rDNA found in plant tissues, five degenerated oligonucleotide primers were designed (and produced upon instructions by Life Technologies, UK) in the conserved regions of the 16S rDNA gene, based on the sequences of 121 different bacteria and algae/plant chloroplast DNA (Table 1), in order to be universal for most bacteria (Table 2 and Figure 1).

**Table 2.** Combinations of primers that detect bacteria in plant tissues of *Billbergia magnifica* ssp. *acutisepalia*.

Pair of primers^a^	Code	Sequences *5′-3′*	Annealing temperature	Size of PCR product	Specificity^b,c,d^
DF11 × DR11	DΔα	BATYAGBTDGTWGGHVRGGT	59 °C	∼570	**W_1,2,5,8,10_, W_3_ (−1, −2), W_6_ (−1, −1), W_7_ (−3, 0), W_9_ (−1, −2), W_11_ (0, −2), W_12_ (0, −1), W_13_ (0, −1), W_14_ (0, −2).**
		RGACTACCAGGGTATCTARKCCTG			This pair of primers would not detect the above bacterial phyla in all plants but would detect bacteria living in algae.
DF11 × DR22	DΔβ	BATYAGBTDGTWGGHVRGGT	58 °C	∼860	**W_1,2,8,10,11,12,13_ W_3_ (−1, 0), W_4_ (−2, 0), W_5_ (0, −1), W_6_ (−1, 0), W_7_ (−3, 0), W_9_ (−1, 0), W_14_ (0, −4).**
		RGSACTBAASCBRACRYYTC			This pair of primers would not detect the above bacterial phyla in all plants and algae.
DF22 × DR22	DΣα	CRAACAGGMYTAGATACCCTG	58 °C	∼320	**W_1,2,8,10_, W_3_ (−2, 0), W_5_ (0, −1), W_6_ (−2, 0),W_7_ (−1, 0), W_9_ (−2, 0), W_11_ (−2, 0), W_12_ (−1, 0), W_13_ (−2, 0), W_14_ (−2, −4).**
		RGSACTBAASCBRACRYYTC			This pair of primers would not detect the above bacterial phyla in all plants and algae.
DF22 × DR33	DΣβ	CRAACAGGMYTAGATACCCTG	59 °C	∼620	**W_1,2,8,10_, W_5_ (0, −1), W_6_ (−2, −1), W_7_ (−1, −1), W_9_ (−2, 0), W_11_ (−2, −2), W_12_ (−1, −1), W_14_ (−2, 0).**
		TGTACAAGVCCCRRGRACRY			This pair of primers would detect all bacterial phyla that live in all plants but not in bacteria which inhabit algae.

^a^With reference to *Escherichia coli* numbering scheme of 16S rDNA gene, the positions of the degenerated primers are: **DF11**: 241–260, **DF22**: 779–799, **DR11**: 806–783, **DR22**: 1098–1079 and **DR33**: 1396–1377.

^b^Based on alignments of 16S rRNA sequences of 70 bacteria belonging to all known phyla, nine strains of bacteria that *cannot* be cultured on nutrient media and chloroplasts from 32 different algae and plants.

^c^According to Woese's classification system [8]: **W_1_**: Proteobacteria, **W_2_**: Green sulphur bacteria, **W_3_**: Green non-sulphur bacteria, **W_4_**: Cyanobacteria, **W_5_**: *Planctomyces–Pirella*, **W_6_**: *Spirochetes*, **W_7_**: *Bacteroides–Flavobacterium*, **W_8_**: *Chlamydia*, **W_9_**: *Deinococcus–Thermus*, **W_10_**: Gram-positive bacteria, **W_11_**: *Aquifex–Hydrogenobacter*, **W_12_**: *Thermotoga–Thermosipho*, **W_13_**: Uncultured bacteria, **W_14_**: Archaea.

^d^The numbers in parentheses show the number of nucleotides which did not match the forward/reverse primers in order to work for those particular phyla.

### Origin of bacterial isolates and plant tissue

Bacterial isolates used for these experiments were a Gram-positive *Bacillus* sp. and a Gram-negative *Pseudomonas* sp. obtained in previous studies [9] and which could be classified by sequencing of their 16S rDNA gene. The plant tissue used for DNA extraction was derived from microbe-free clonal *in vitro* plants of *B. magnifica* ssp. *acutisepalia* produced by regeneration of plants via somatic embryogenesis.[7] The latter plants did not yield any detectable and culturable microbes following extensive and rigorous enrichment plating tests.[9]

### Estimation of the number of bacterial cells

Three different methods were used to estimate the number of bacterial cells present per inoculum. Ten-millilitre aliquots of Trypto-Soya Broth medium (Oxoid, Unipath Ltd) were inoculated with single bacterial colonies and incubated overnight in an orbital shaker (Orbital mixer, Denley Ltd, UK) at 200 r/min under room temperatures (25 ± 3 °C). Bacterial cells were washed twice with 10 mmol/L MgCl_2_ and were resuspended in the same buffer; then, the absorbance of the suspensions was adjusted to an optical density of 1 (at 600 nm), using a spectrophotometer (SP8-100 UV/VIS, Pye/ATI Unicam/Philips, Spectronic Camspec Ltd, UK).

To estimate the number of bacterial cells in each suspension, three 100 μL aliquots were plated onto Trypto-Soya Agar (TSA) media [30 mL on each (9 cm) Petri dish (Sterilin, UK)] using a sterile glass loop to spread each aliquot evenly over the media. The plates were incubated at 25 °C for 24 h and single colonies counted in order to estimate the number of bacterial cells in the suspension. Estimation of the bacterial cells was also made by the counting chamber method (Weber Scientific International Ltd, England). A drop of suspension, with an estimated OD_600_ of 0.1, was placed on the slide, and bacterial cells were counted under a dissecting microscope (Dialux 20 EB, Ernst Leitz Wetzlar GMBH, Germany). Following this standardization, 10-fold dilutions were then derived (Table 3).

**Table 3.** Estimation of bacterial cell densities used as inoculums.

	Gram^+^*Bacillus* isolate			Gram^−^*Pseudomonas* isolate		
Methods	α_1_	β_1_	γ_1_	α_2_	β_2_	γ_2_
Plating on TSA medium^a^	4.15 × 10^6^	4.15 × 10^5^	4.15 × 10^4^	6.33 × 10^6^	6.33 × 10^5^	6.33 × 10^4^
	SE ± 2.861			SE ± 3.305		
Spectrophotometry at OD_600 nm_^b^	2.50 × 10^7^	2.50 × 10^6^	2.50 × 10^5^	2.50 × 10^7^	2.50 × 10^6^	2.50 × 10^5^
Counting chamber^c^	2.67 × 10^8^	2.67 × 10^7^	2.67 × 10^6^	1.02 × 10^9^	1.02 × 10^8^	1.02 × 10^7^
	SE ± 1.289			SE ± 3.28		

Note: Bacterial suspensions (50 μL) used as inoculum in 10-fold dilutions α_1_, β_1_, γ_1_ of a Gram-positive *Bacillus* sp. isolate and α_2_, β_2_, γ_2_ of a Gram-negative *Pseudomonas* sp. isolate. SE: standard error.

^a^Colony-forming units cfu/50 μL of bacterial suspension.

^b^Absorbance of bacterial cell suspensions at 600 nm with OD1 corresponds to ca. 5 × 10^8^ cells per mL of suspension.[12]

^c^Bacterial cell suspensions.

### Sample preparation prior to DNA extraction

Samples of 50, 250 and 500 mg of plant tissue were weighed on a digital top-pan balance (Sartorius, Fisher Ltd, UK) and placed into 1.5 mL sterile microtubes. A total of 50 μL of bacterial inoculum was added to each microtube after the plant tissue had been homogenized in the buffer being tested using a plastic sterile pestle. Bacterial inoculums were prepared in serial 10-fold dilutions (α_1_, β_1_, γ_1_) for Gram-positive and (α_2_, β_2_, γ_2_) for Gram-negative bacteria, after estimating the number of bacteria present in the stock suspension using 10 mmol/L MgCl_2_.

### Nucleic acid preparation

Three different DNA extraction methods were tested for their capacity to extract nucleic acids from bacteria/plant mixtures, from pure cultures of bacteria and from non-contaminated *in vitro* plant tissue. The latter samples were used also as negative controls for the PCR reactions.

The first protocol was the *MiniPrep* method [10] and was based on chemical disruption of cells, using mercaptoethanol following isoamyl alcohol/chloroform separation. This protocol was utilized for contaminated plant samples, which were maintained previously in a freezer; mortars and pestles were thoroughly cleaned and kept in a freezer (at least overnight at −20 °C). Contaminated plant samples were homogenized using liquid nitrogen and 150 mg of the homogenized tissues was transferred into a new 1.5 mL sterile microcentrifuge tube. The next steps involved: (1) addition of 600 μL DNA extraction buffer [3% (w/v) cetyl trimethylammonium bromide (CTAB), 1.4 mol/L NaCl, 20 mmol/L ethylenediaminetetraacetic acid (EDTA) pH 8.0, 100 mmol/L Tris–HCl pH 8.0 and 1% (w/v) polyvinylpyrrolidone PVP-40T (soluble)] and mixing well by vortexing for 30 s, (2) addition of 20 μL 2-mercaptoethanol and mixing well by vortexing for 30 s, (3) incubation of the homogenized samples at room temperature for 10 min, (4) addition of 250 μL chloroform/isoamyl ethanol, 24:1 (v/v) and mixing very well by vortexing for 30 s, (5) phase separation by microcentrifugation (at 3380*g*) for 15 min, (6) transfer of supernatant in a new 1.5 mL sterile microcentrifuge tube, (7) addition of 250 μL chloroform/isoamyl ethanol, 24:1 (v/v) and mixing very well by vortexing for 30 s, (8) phase separation by microcentrifugation (at 3380*g*) for 10 min, (9) transfer of supernatant in a new 1.5 mL sterile microcentrifuge tube and an estimate made of the total volume of supernatant, (10) addition of 2.5 volumes of 100% ethanol into the volume of supernatant (step 9), (11) mixing gently and sample incubation at room temperature for 90 min, (12) microcentrifugation (at 17,100*g*) for 10 minutes, (13) supernatant removal, addition of 500 μL of 70% ethanol and vortexing thoroughly for 1 min, (14) microcentrifugation (at 17,100*g*) for 3 min and removal of ethanol, (15) air-dry the pellet for 10 min at 37 °C, (16) resuspension of the pellet in 50–100 μL of molecular grade water or Tris–EDTA (TE) buffer, (17) storage of DNA solution at −20 °C.

The second method was the protocol of Lawson et al. [11] for bacterial DNA extraction; this involved disrupting cells with an enzymatic procedure followed by phenol/chloroform separation. The third method tested was the RapidPrep^®^ Micro Genomic DNA Isolation Kit for cells and tissue [12] which involved isolating DNA by means of anion-exchange chromatography in a spin-column format.

### PCR amplification

The PCR mixtures (50 μL) contained 5 μL of 10× reaction buffer [16 mmol/L (NH_4_)_2_SO_4_; 70 mmol/L Tris–HCl pH 8.8; 0.1% Tween 20]; 0.06 μL of each deoxynucleoside triphosphate (100 mmol/L); 0.15 μL of BioTaq (5 u/μL; *Thermus aquaticus*; Bioline); 1.5 μL MgCl_2_ (50 mmol/L); 2 μL of each degenerated primer (30 pmol/L); 1 μL template DNA (approximately 10–40 ng); and 38.11 μL of analytical grade water. PCR amplification was performed with a thermal cycler (GenAmp In Situ PCR System 1000, PerkinElmer Cetus); cycles consisted of 6 min denaturation at 96 °C followed by 40 cycles of 30 s at 95 °C for further denaturation, 15 s at 59 °C (for DΣβ) primer set annealing, 1 min at 72 °C extension and ended with a 10 min extension at 72 °C. The annealing temperature for the other set of primers is shown in Table 2.

After amplification, 6 μL of DNA loading buffer (20% glycerol, 5 mmol/L EDTA, bromophenol blue) was added and 16 μL of this mixture was separated by electrophoresis in a 1.5% agarose gel in Tris–acetate–EDTA electrophoresis buffer (pH 8.5) at room temperature. Gels were stained for 20 min in ethidium bromide (0.75 μg/L in deionized water), distained (5 min in deionized water) and viewed on a UV transilluminator.

## Results and discussion

**Figure 2.**
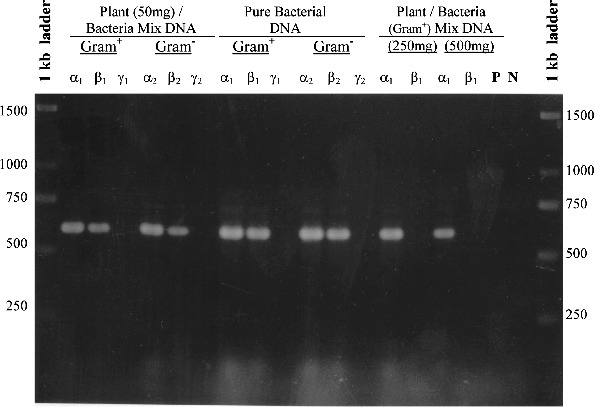
Detection of bacterial DNA in plant tissues, using the DΣβ pair of primers (Table 2). Different DNA templates were tested for both Gram-positive and Gram-negative bacteria used as inoculum with either 50, 250 or 500 mg *in vitro* tissue of *Billbergia* and extracted by the *MiniPrep* method.[10] P is the control using pure plant DNA as a template and N is the negative control with no DNA template; α_1_, β_1_, γ_1_ and α_2_, β_2_, γ_2_ are different concentrations of bacterial cells used as inoculum (Table 3).

**Figure 3.**
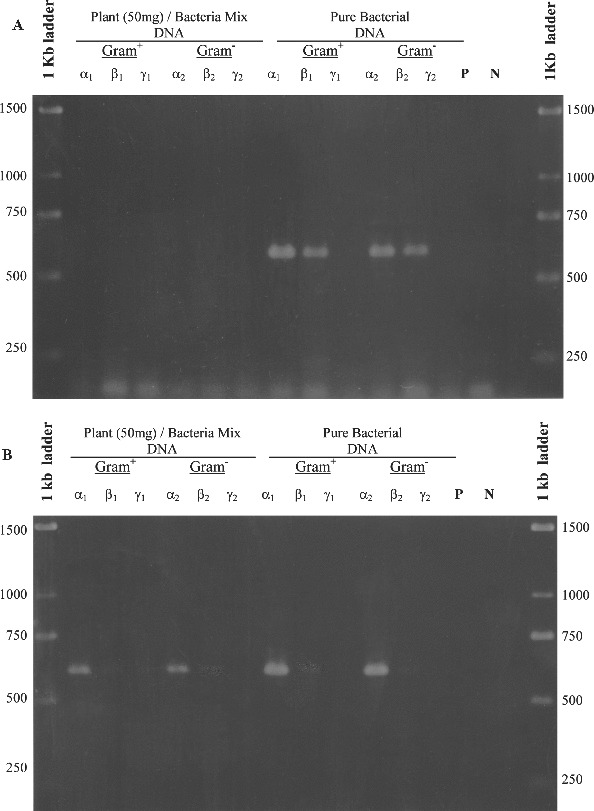
Detection of bacterial DNA in plant tissues, using the DΣβ pair of primers (Table 2) that amplify an approximately 620 bp fragment in the 16S rDNA gene of Gram-positive and Gram-negative bacteria present in *Billbergia* tissues. DNA extracts used as template for PCR amplification obtained by (A) Lawson's method for extraction of bacterial genomic DNA [11] and (B) RapidPrep^®^ Micro Genomic DNA Isolation Kit for cells and tissues. P is the control using pure plant DNA as a template and N is the negative control with no DNA template; α_1_, β_1_, γ_1_ and α_2_, β_2_, γ_2_ are different concentrations of bacterial cells used as inoculum (Table 3).

Two forward and three reverse oligonucleotide primers were designed (Table 2) on the basis of a total of 121 different published 16S rDNA sequences derived from eubacteria, archae and chloroplasts of algae and plants. Using bacterial DNA from a *Pseudomonas* sp. isolate and plant genomic DNA from *in vitro* plants of *Billbergia*, combinations of the degenerated primers amplified four different fragments in the 16S rDNA gene of bacteria, without amplifying chloroplastic DNA (Figure 1). The extent to which plant/bacteria mix lysates can be amplified is largely dependent on the method used to extract DNA. Enzymes and inhibitors in the lysate can have a significant, deleterious effect on the efficiency and sensitivity of PCR assays.[13] For this reason, three different DNA extraction procedures were compared to test their effects on PCR amplification using the DΣβ pair of primers. The results of PCR efficiency and sensitivity using DNA templates from each DNA extraction method showed that the best level of detection was achievable at approximately 2.5 × 10^6^ bacterial cells per 50 mg *in vitro* tissue of *Billbergia*, for both Gram-positive and Gram-negative bacteria (Figure 2). The *MiniPrep* extraction method [10] produced a strong PCR amplification fragment (Figure 2), compared to the faint band produced by DNA extracted by the RapidPrep^®^ method (Figure 3(B)) and the absence of a band for DNA extracted by Lawson's method (Figure 3(A)). The sensitivity of the *MiniPrep* method in different ratios of bacteria/plant tissue used for DNA extraction was examined. A total of 2.5 × 10^7^ bacterial cells in 500 mg *in vitro* tissue of *Billbergia* were detected but the template DNA did not produce as strong an amplification product as those in which either 50 or 250 mg plant tissue was used. Generally the lesser the plant tissue used for DNA extraction, the lower the number of bacterial cells detected (Figure 2).

Micropropagation of the epiphytic bromeliad *B. magnifica* ssp. *acutisepalia* from vegetative explants is difficult and in many cases impossible due to heavily contaminated explant material used to initiate *in vitro* cultures.[7] Our previous studies showed that many different contaminant bacterial species could be recovered despite the fact that harsh chemical surface sterilization treatments were used.[9] In order to study the microcosmos of *Billbergia* and the interactions of those microbes with the plant during the micropropagation and post-weaning processes, we developed a PCR-based bacterial detection tool.

The degenerated primers designed in this study were based on the sequences of the 16S rDNA gene belonging to all bacterial phyla and most of the known bacterial families available in the Gene Bank/EMBL (European Molecular Biology Laboratory) databases. Most of the 16S rDNA gene sequences of plant chloroplasts that were available in the Gene Bank/EMBL databases showed a high degree of similarity. The sequence of the 16S rDNA gene of *Billbergia* was also very similar to other plants listed in the databases and the conserved regions where the degenerated primers designed were almost identical. Thus, the degenerated primers designed for *Billbergia* would appear to have the potential to be used for other plants as well.

The molecular detection of bacterial contaminants in plant tissue cultures indicates that: (1) specific oligonucleotide primers (at genus, family or even at phylum level) can be designed and can detect bacteria present not only in tissues of *B. magnifica* ssp. *acutisepalia* but in other plants as well; (2) primers can amplify members of culturable and unculturable bacterial and archaeal groups (Tables 1 and 2); (3) the isolation protocol used for the extraction of genomic DNA affects the template purity and most importantly the efficiency of the PCR reactions, as previously shown by Simon et al. [14] and in the course of the present study (Figures 2 and 3). The generated PCR product can be subsequently used for identification of the bacteria harboured in the plant tissues, by cloning and sequencing.

## Conclusions

The results of the present study showed that specific oligonucleotide primers (at genus, family or even at phylum level) can be designed and used for molecular detection of bacterial contaminants not only in tissue cultures of *B. magnifica* ssp. *acutisepalia* but in other plants as well. A set of primers that can amplify members of culturable and unculturable bacterial and archaeal groups was proposed. The isolation protocol used for the extraction of genomic DNA was observed to affect the template purity and most importantly the efficiency of the PCR reactions. The generated PCR product can be subsequently used for identification of the bacteria harboured in the plant tissues, by cloning and sequencing.
